# Stabilization of the VO_2_(M2) Phase and Change in Lattice Parameters at the Phase Transition Temperature of W_*X*_V_1–*X*_O_2_ Thin Films

**DOI:** 10.1021/acsami.3c11484

**Published:** 2023-10-24

**Authors:** Artitsupa Boontan, Eric Kumi Barimah, Paul Steenson, Gin Jose

**Affiliations:** † School of Chemical and Process Engineering, University of Leeds, Clarendon Road, Leeds LS2 9JT, U.K.; ‡ School of Electronic and Electrical Engineering, University of Leeds, Clarendon Road, Leeds LS2 9JT, U.K.

**Keywords:** fs-PLD, vanadium dioxide, M1 and M2 phases, doping, W, phase transition

## Abstract

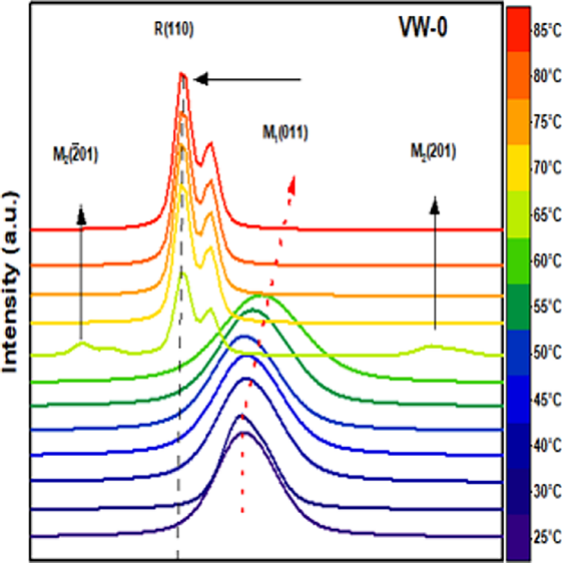

Various methods have been used to fabricate vanadium dioxide (VO_2_) thin films exhibiting polymorph phases and an identical chemical formula suited to different applications. Most fabrication techniques require post-annealing to convert the amorphous VO_2_ thin film into the VO_2_ (M1) phase. In this study, we provide a temperature-dependent XRD analysis that confirms the change in lattice parameters responsible for the metal-to-insulator transition as the structure undergoes a monoclinic to the tetragonal phase transition. In our study, we deposited VO_2_ and W-doped VO_2_ thin films onto silica substrates using a high repetition rate (10 kHz) fs-PLD deposition without post-annealing. The XRD patterns measured at room temperature revealed stabilization of the monoclinic M2 phase by W^6+^ doping VO_2_. We developed an alternative approach to determine the phase transition temperatures using temperature-dependent X-ray diffraction measurements to evaluate the *a* and *b* lattice parameters for the monoclinic and rutile phases. The *a* and *b* lattice parameters versus temperature revealed phase transition temperature reduction from ∼66 to 38 °C when the W^6+^ concentration increases. This study provides a novel unorthodox technique to characterize and evaluate the structural phase transitions seen on VO_2_ thin films.

## 1 Introduction

Over the past few decades, numerous polymorph phases of vanadium dioxide (VO_2_) thin films with an identical chemical formula, such as VO_2_(M1), VO_2_(M2), VO_2_(R), VO_2_(A), VO_2_(B), VO_2_(C), VO_2_(D), VO_2_(P), VO_2_(R), and VO_2_(T), have been fabricated, and their properties were studied.^1,2^ The formation of various V–O systems can be attributed to different V and O atom sites in the crystalline lattice of coordination polyhedral.^3^ These polymorph phases can be transformed into other phases under certain conditions and with different transition temperatures.^1^ For instance, VO_2_ (D) can undergo a VO_2_(R) phase transformation at a transition temperature of ∼320 °C, while VO_2_(A) and VO_2_(B) to VO_2_(R) phase transition temperatures occur at 475 °C.^1^ However, the phase transitions of these polymorphs are not reversible by either reducing or rising temperature due to changes in the structural and immense strain or stress transformation. On the other hand, the monoclinic VO_2_(M1) phase has been studied extensively during the last few decades because it undergoes an abrupt metal–insulator transition (MIT) at ∼68 °C,^2,3^ which is reversible by altering the temperature, the electrical field, incident illumination, and pressure strain properties. Such reversible phase transition temperatures are associated with structural modification from a low-temperature monoclinic M1-phase to a high-temperature rutile R-phase.^3^ An intermediate VO_2_(M2) polymorph phase with a β-angle of 91.88° could be stabilized at room temperature or exist during the MIT from M1→ M2 → R. This can be achieved by doping with a low concentration of various transition elements, such as W, Al, etc., and introducing strain in the film.^4,5^

Meanwhile, VO_2_(M1) has a phase transition temperature slightly higher than room temperature, arguably limiting its practical applications. As a result, several fabrication techniques have been adopted to reduce the VO_2_(M1) phase transition temperature to near room temperature by doping with high-valent transition metals such as Al^3+^, Ta^5+^, Mo^6+^, Nb^5+^, and W^6+^.^5−11^ Alternatively, to doping VO_2_ with transition metals; the phase transition temperature can be controlled by changing particle sizes, surface morphologies, and crystalline phases during the competitive nucleation growth mechanism.^9^ On the other hand, doping with high-valent transition metals of the VO_2_ thin films leads to lattice distortion and thus induces local stress and strain, which have been shown to reduce the phase transition temperature.^10−14^ Thus, lowering the phase transition temperature of VO_2_ thin films is attractive for numerous applications such as IR uncooled bolometers, thermochromic coatings, optical switching devices, ultrafast switching, smart radiator devices for spacecraft, and Mott transition field effect transistor.^3,6,7^ Recently, there have been a few studies on pure VO_2_(B) and VO_2_(M1) materials, focusing on structural unit cell *a*, *b*, and *c* lattice parameters to determine the MIT behavior and transition temperature.^15,16^

Different techniques have been implemented to fabricate VO_2_ and transition metal-doped VO_2_ thin films, aiming to lower their transition temperatures, which include sputtering, hydrothermal, nanosecond laser (ns) PLD,^9−11^ RF-magnetron sputtering, and femtosecond (fs) PLD.^3,17^ Chen et al.^11^ synthesized Al^3+^-doped VO_2_ thin film onto silicon and soda-lime substrates using Al-doped V_2_O_5_ target and ns- PLD with a KrF excimer laser at a wavelength of 248 nm. A transition temperature of 40 °C was reported for VO_2_ doped with an Al^3+^ thin film compared to 67 °C for the pure VO_2_ thin films. Similarly, VO_2_ and W_*x*_V_1–*x*_O_2_ thin films deposited were fabricated with a reactive pulsed laser deposition and a XeCl excimer ns-laser at a wavelength of 308 nm by Soltani et al.^12^ They observed a transition temperature of about 36 and 68 °C for W-doped VO_2_ and VO_2_ thin films, respectively.

In this study, we fabricated the VO_2_ and W_*x*_V_1–*x*_O_2_ thin films onto a silica substrate without post-annealing using femtosecond pulsed laser deposition at a repetition rate of 10 kHz. We systematically investigated the crystal structure of the VO_2_ and W_*x*_V_1–*x*_O_2_ films by using TEM and XRD patterns. In addition, the FullProf Software was utilized to analyze the temperature-dependent XRD pattern data to evaluate the *a* and *b* lattice parameters and to predict the phase transition temperatures of these samples.

## 2 Experimental Methods

### 2.1 Sample Preparation and Fabrication

Vanadium pentoxide (V_2_O_5_) and W^6+^-doped vanadium pentoxide (V_2_O_5_) targets with the molar composition of (100 – *x*) V_2_O_5–_*_x_*WO_3_– (*x* = 0, 0.5, 1.0, and 1.5 mol %, namely, VW0, VW1, VW2, and VW3) were prepared. High-purity V_2_O_5_ (≥99.99%) and WO_3_ (99.99%) materials were purchased from Alfa Aesar. About 25 g batch of pure V_2_O_5_ power and the appropriate amount of WO_3_ and V_2_O_5_ powers were weighed to prepare W-doped V_2_O_5_ powder material. The WO_3_ and V_2_O_5_ powders were thoroughly mixed using a mortar and a pestle until a homogeneous mixture was obtained. Each powder sample was pressed into a pallet (PLD target) with dimensions of 30 mm × 40 mm × 2 mm using a Spec press with a 1 t load for 5 min. An ultrasonic bath was used to clean the 20 mm, 30 mm × 1 mm silica substrates at 50 °C followed by an acetone and isopropyl alcohol rinse and dried with a high-purity nitrogen gas-gun. The substrate and the target were mounted into respective holders within the PLD chamber. The PLD chamber was then evacuated to a base pressure of 10^–7^ Torr before backfilling to a working pressure of 70 mTorr using high-purity process oxygen (99.99%). The separation distance from the substrate to the target was kept at 60 mm, and the substrate temperature was maintained at 700 °C. Pure V_2_O_5_ and W-doped V_2_O_5_ targets were ablated to deposit thin films with a KMLabs Wyvern 1000–10 solid-state Ti:sapphire laser/amplifier and a laser fluence of 0.27 J/cm^2^ at a 75 kHz repetition rate. The total deposition time was in the region of 2 h.

### 2.2 Characterization

The surface topography was examined and recorded using a Carl Zeiss EVO MA15 scanning electron microscopy (SEM). Following the SEM imaging, ImageJ software was utilized to determine isolated particle distribution deposited on the substrate. A focused ion beam (FIB) (FEI Helios G4 CX DualBeam) machine was employed to prepare an in situ TEM cross section of each thin film. The FEI Tecnai TF20 transmission electron microscope fitted with a HAADF detector was utilized to acquire cross-sectional images, together with high reflectance TEM images and selected area electron diffraction (SAED) patterns. The room temperature X-ray diffraction patterns of the as-prepared samples were recorded using a P’Analytical X’Pert diffractometer (Cu Kα_1_ radiation = 1.54056 Å) at 45 kV and 40 mA. The XRD patterns were measured from 10 to 60° with a step size of 0.02 for angle 2θ. Subsequently, the temperature-dependent studies of XRD patterns were collected using a Malvern P’Analytical Empyrean Diffractometer (Cu*K*α_1_ radiation = 1.54056 Å) system equipped with an Anton Parr HTK1200 heating stage unit. The temperature dependent XRD data was recorded in the temperature ranging from 10 to 80 °C with an increment of 5 and 10 °C. Each sample was mounted on an Anton Parr HTK1200 heating stage with housing, then heated to the appropriate temperature and kept for 5 min to stabilize before XRD data was collected. The XRD measurements of the VO_2_ and W_*x*_V_1–*x*_O_2_ thin films were analyzed using the FullProf Suite software 3.00, and the pseudo-Voigt profile function for Profile matching and Rietveld refinement were performed. The *a* and *b* lattice parameters of monoclinic and rutile VO_2_ phases were tracked and evaluated at different temperatures using Le Bail analysis to determine the phase transition temperature. The X-ray photoelectron spectra (XPS) were recorded on an Omicron energy analyzer (EA-125) with an Al Kα (1486.6 eV) X-ray source. Temperature-dependent resistivity measurements data were performed from 25 to 100 °C for heating and cooling using the Ossila Four-Point Probe (Ossila Ltd., Sheffield, UK).

## 3 Results and Discussion

### 3.1 Surface Morphology

**Figure 1:**
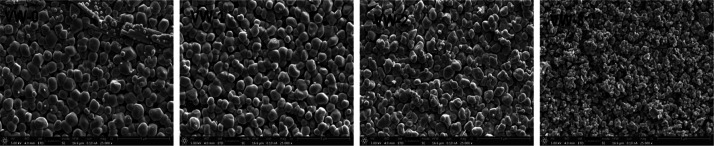
Surface morphology of top-view SEM images of the undoped and W-doped VO_2_ thin films.

**Figure 2:**
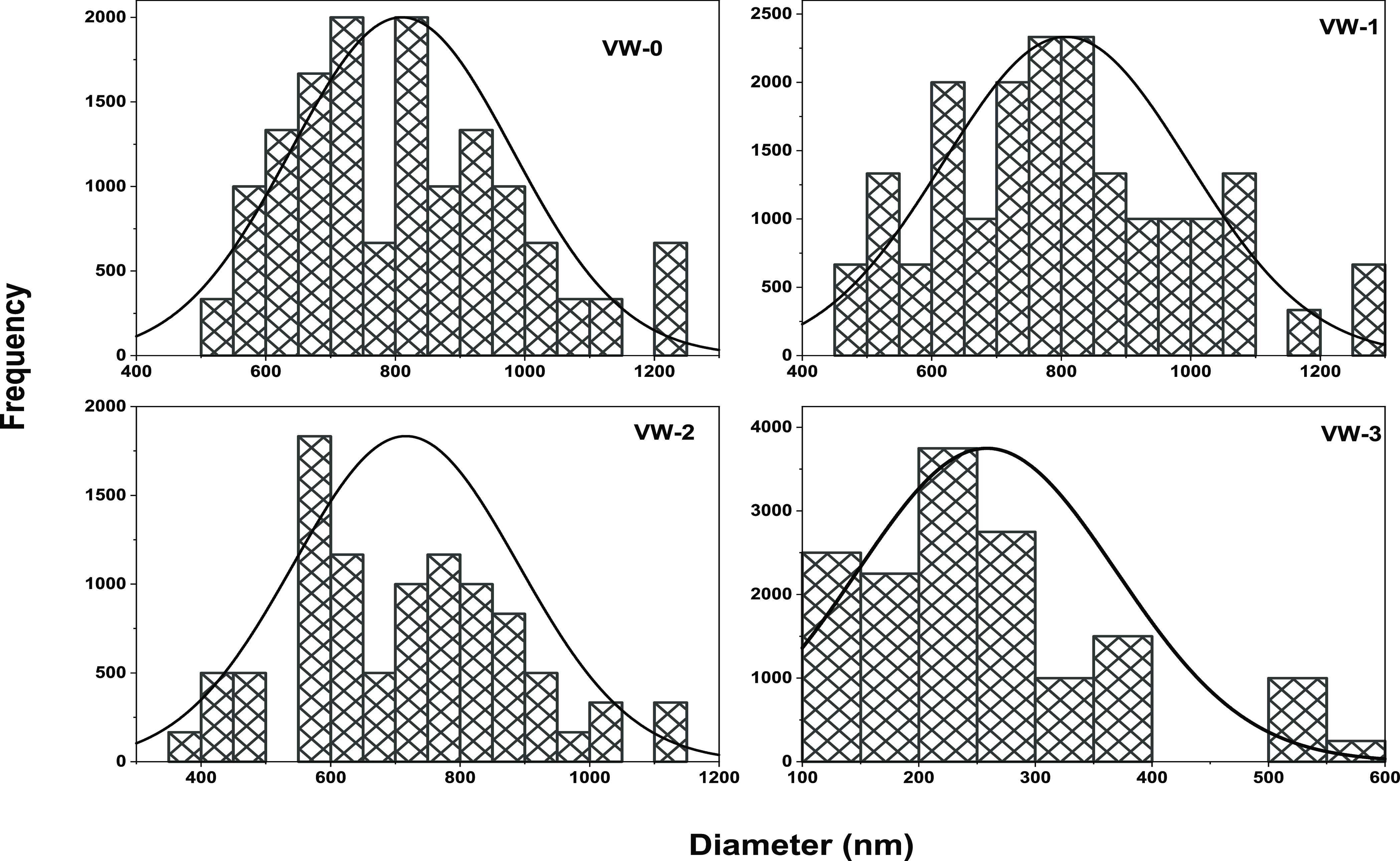
Histogram showing the particle size distribution of undoped and W-doped VO_2_ thin films for samples VW-0, VW-1, VW-2, and VW-3.

The SEM image analysis was initially acquired to understand the effect of doping W with VO_2_ on the morphology and grain sizes. Figure 1 shows the top-view SEM images and particle size distribution of the VO_2_ and different concentrations of W_*x*_V_1–*x*_O_2_ thin films deposited on a silica substrate labeled VW-0, VW-1, VW-2, and VW-3. Noticeably, the particle sizes are uniform with irregular and spherical shapes for samples VW-0, VW-1, and VW-2. However, as the W^6+^ ion concentration increased, the grain sizes decreased immensely for the sample VW-3. The decrease in grain size, surface porosity, and electronic structure of sample VW-3 may be attributed to the crystal lattice’s energetic and kinetically disordered crystallization.^18^ In addition, substituting the W^6+^ ion into the VO_2_ lattice crystal may deform the matrix’s bonding lengths and coordination spheres, leading to interfacial strain and decreasing grain size. Subsequently, the VO_2_ particle distribution on the silica substrate was evaluated using ImageJ software and SEM images. Figure 2 shows a particle size histogram fitted with Gaussian distribution curves. These analyses reveal average particle sizes of 800 ± 20, 800 ± 23, 700 ± 50, and 200 ± 17 nm for samples VW-0, VW-1, VW-2, and VW-3.

### 3.2 TEM Cross Section and Crystallography Analysis of the Thin Films

**Figure 3:**
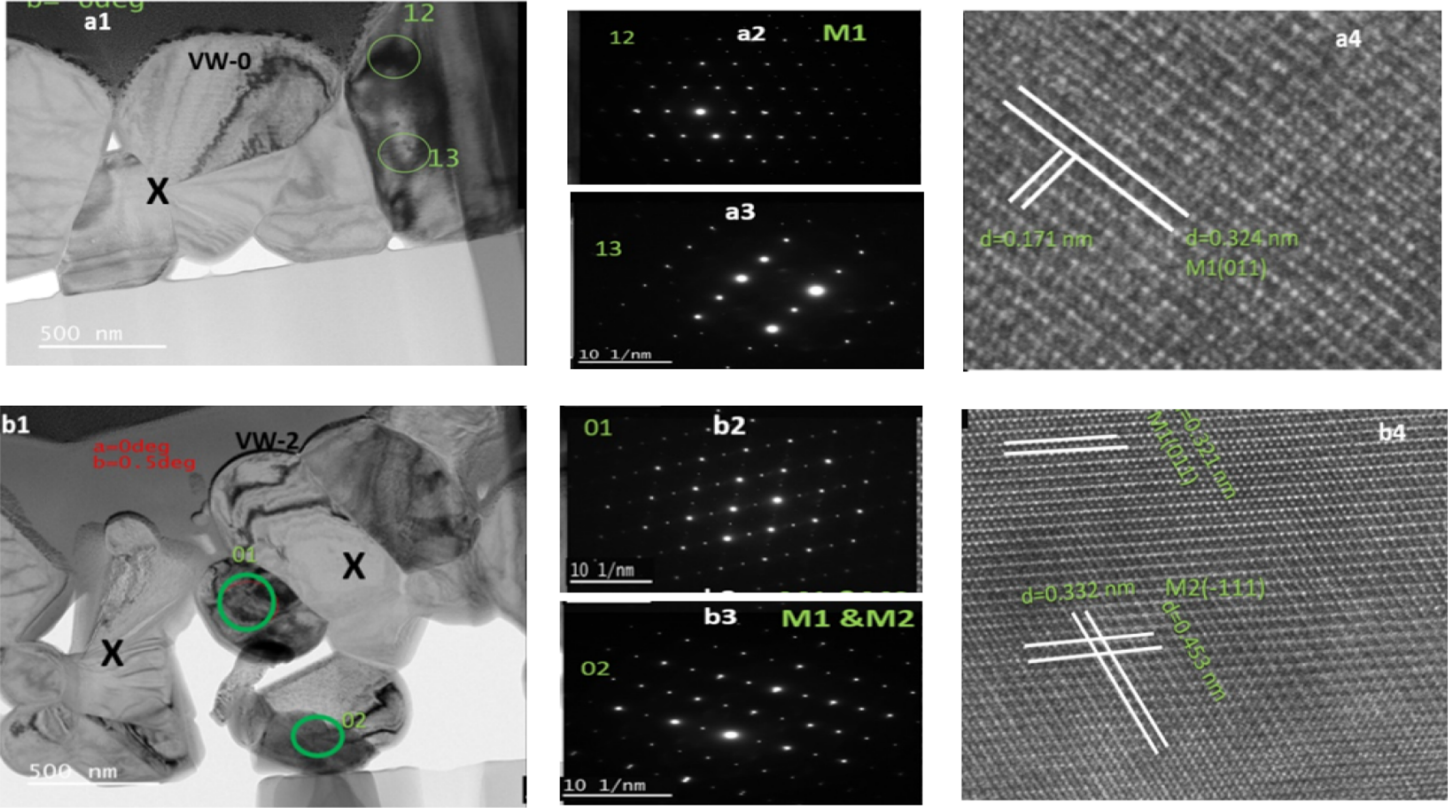
(a1, b1) TEM cross-sectional image of samples VW-0 and VW-2; (a2, a3, b2, b3) corresponding SAED patterns of two different areas; (a4, b4) HRTEM images for resolving the VO_2_ crystal lattices.

**Figure 4:**
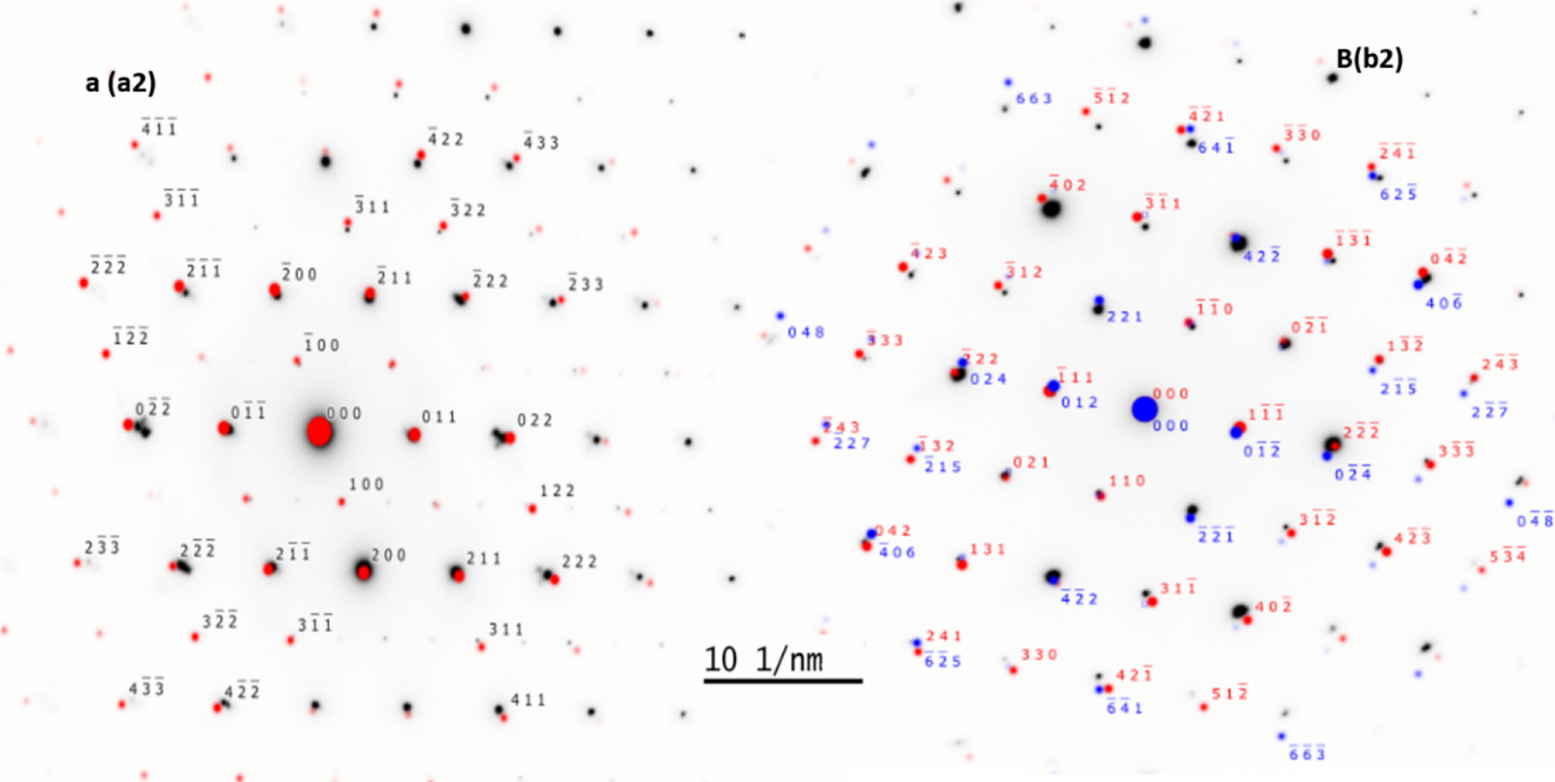
Electron diffraction patterns of the VO_2_ matrix from the [011] plane of the VO2 thin film. (a) Sample VW-0 of Figure 3a2 and (b) sample VW-2 of Figure 3b2.

Bright-field TEM cross-sectional images of all the fabricated samples were prepared using a focused ion beam (FIB, FEI Helios G4 CX DualBeam). Figure 3a1,b1 shows bright-field cross-sectional TEM images of the samples VW-0 and VW-2 exhibiting heterostructures with average thin film thicknesses of ∼0.98 and ∼1.06 μm. The SAED patterns were acquired randomly from the areas circled in green, as shown in Figures 3a2,a3,b2,b3. The SAED pattern depicted in Figure 3a2,a3 demonstrates the basic structural information on the monoclinic VO_2_ (M1) phase without impurity. The SAED pattern also reveals the characteristics of long-range ordered polycrystalline structures. Furthermore, the magnified HRTEM image has interplanar spacing correlating to an out-of-plane and in-plane spacings of 0.342 and 0.171 nm, which correspond to (110) and (−211) planes of VO_2_ (M1) as illustrated in Figure 3a4. Similarly, the SAED pattern of the sample VW-2 shows mixed monoclinic M1 and M2 phases of VO_2_ along with interplanar spacings of 0.321 and 0.453 nm, which correspond to (110) and (−111) zone axis. To validate the crystal structure of the thin films prepared, electron diffraction patterns of samples VW-0 and VW-2 SAED were employed to determine lattice parameters using SingleCrystal software for comparison. Figure 4a shows two mirror lattice constant patterns obtained from sample VW-0, which confirms the M1 phase of the VO_2_ polycrystalline lattice. Likewise, Figure 4b, consisting of Figure 3b2, shows the lattice constant patterns acquired from the VO_2_ lattice along the [110] and [−111] plane axes for the M1 and M2 mixed phases indicated in red and blue.

### 3.3 Structural Transformation and Stability of the M2 Phase

**Figure 5:**
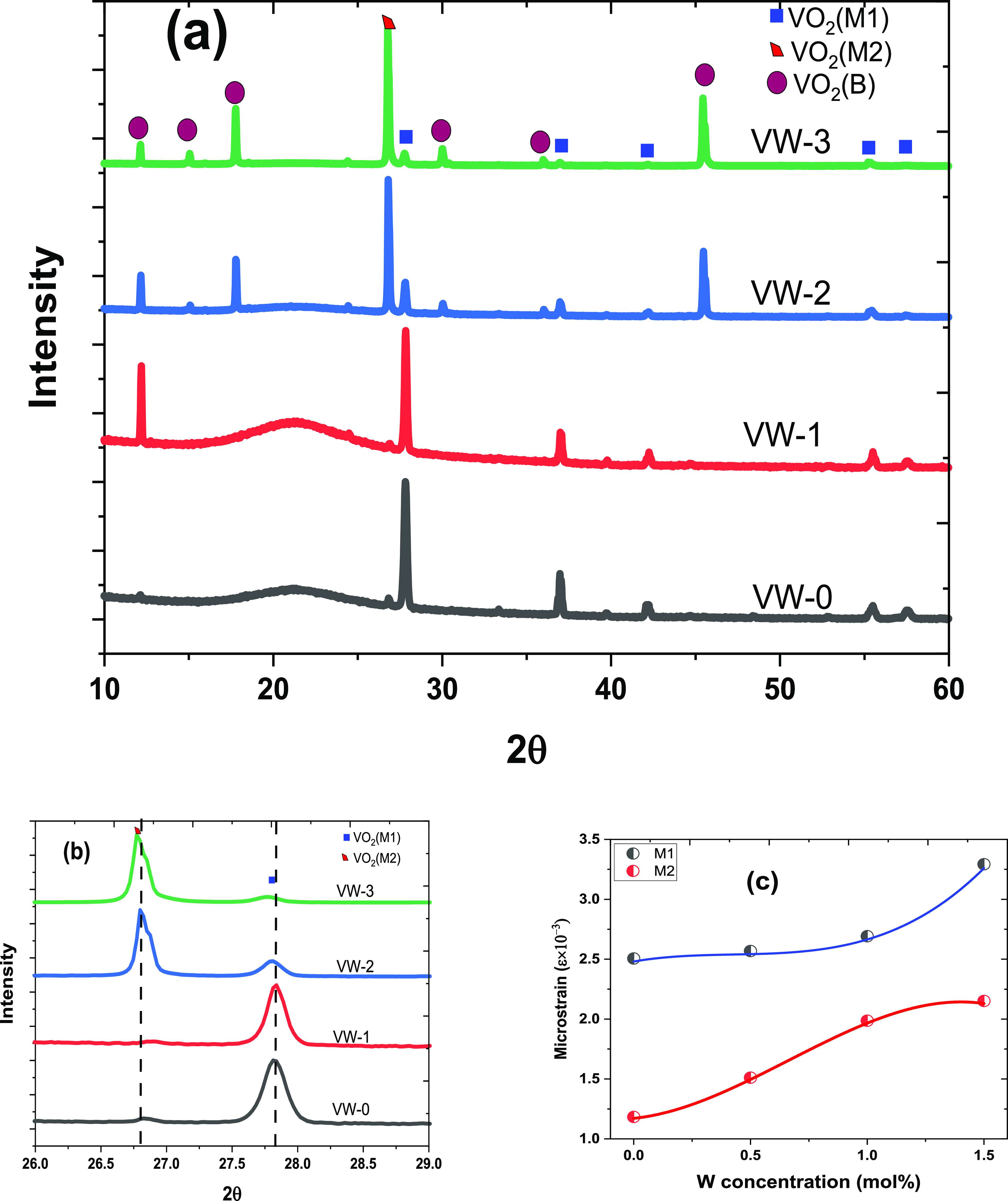
(a) XRD patterns of the undoped VO_2_ and different concentrations of W-doped VO_2_ thin films at room temperature at θ–2θ scans showing VO_2_(M1), VO_2_(M2), and VO_2_(B) phases, (b) XRD patterns for θ–2θ scans ranging from 26° to 29.0°, and (c) variation of microstrain with W concentration.

Following the SEM analysis, XRD patterns of the fabricated undoped VO_2_ and W_*x*_V_1–*x*_O_2_ thin films were collected using a θ–2θ scan to investigate their crystalline phases, as depicted in Figure 5. The diffraction patterns of the undoped VO_2_ (sample VW-0) illustrate about seven prominent polycrystalline peaks centered at 2θ = 27.82°, ∼33.37°, ∼37.04°, ∼39.04°, ∼42.14°, ∼ 55.43°, and ∼57.8°. These peaks corresponded to the following crystallographic planes (*hkl*) of (011), (−102), (200), (−112), (210), (220), and (022), with the reflection of VO_2_ (M1) phase and crystal group of P21/c (JCPDS Card No. 72- 0514).^19^ The XRD patterns obtained for undoped VO_2_ thin film structures are comparable to polycrystalline structures reported in the literature.^1^ Furthermore, the XRD patterns of various concentrations of W^6+^-doped VO_2_ thin film samples exhibit additional orientation peaks at 12.22°, 15.01°, 17.86°, 30.13°, 35.80°, and 45.44° with increasing intensity as the W^6+^ content increases. These different diffraction peaks seen in Figure 5 for samples VW-1, VW-2, and VW-3 are indexed as mixed phases of the monoclinic crystalline phase of VO_2_(M2) and VO_2_(B) with a space group *C*2/*m*, which correlate with the JCPDS 70–3131^2^ and JCPDS Card No. 65–7960.^2,20^ These results confirm the formation of mixed phases consisting of VO_2_(M1), VO_2_(M2), and VO_2_(B) phases of chemical formula W_0.6_V_2.4_O_7_ under the current experimental condition. Figure 5b shows 2θ scan XRD diffraction patterns for VO_2_(M2) and VO_2_(M1) peaks centered at ∼26.80° (−111) and ∼27.82° (011) with the intensity of the M2 phase increasing as W^6+^ content increases. These results indicate that the M2 phase becomes more stable and dominant over the M1 phase as the W^6+^ content increases. This is attributed to the induced microstrain caused by substituting W^6+^ ions into the VO_2_ lattice structure. The XRD patterns agree with the TEM SAED patterns depicted in Figure 4a.

Furthermore, the average crystalline size, *d*, of the four different VO_2_ films fabricated was determined employing the full-width-half-maximum (fwhm) values obtained from diffraction peaks at ∼26.80° and ∼27.82° and Debye–Scherrer equation.^21^
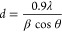
1

The variation in the crystallinity size at the two diffraction peaks for each sample was approximately the same. Nevertheless, the average crystalline size obtained from the Debye–Scherrer equation was <200 nm compared to the average particle size calculated from the SEM images depicted in Figure 2.

Following the Debye–Scherrer equation analysis, the microstrain distortion induced by W^6+^ ions in VO_2_ thin films was determined. The microstrain or strain effect plays an important role in the electrical and optical properties and transition temperatures of the VO_2_ thin films. Therefore, the diffraction peaks centered at 2θ = ∼26.80° (M2) and ∼27.82° (M1) were used to investigate the microstrain or strain effect by following the relationship.^22^
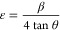
2where λ is the wavelength of the incident X-ray beam (λ = 0.15406 nm), β represents the fwhm, and θ indicates Bragg’s angle.

Figure 5c shows the effect of the microstrain through an increase in W^6+^ content-doped VO_2_ thin films. It was observed that the microstrain increased slightly with W^6+^ content, which may be attributed to the defect induced by W^6+^ in the VO_2_ lattice structure. Thus, such an increase in microstrain may be ascribed to local structure modification of electron–electron interactions in the VO_2_ thin film crystal structure, resulting in stabilization of the M2 phase.^23−25^ Furthermore, the dominating of the M2 phase over the M1 stage at higher W^6+^ content may be ascribed to the differences in visible grain orientation and breaks up of the V^4+^–V^4+^ bonds to form new bonds such as V^4+^–W^6+^, V^3+^–W^6+^, and V^3+^–V^4+^.^23^

### 3.4 Valence States and Ratios of Vanadium

**Figure 6:**
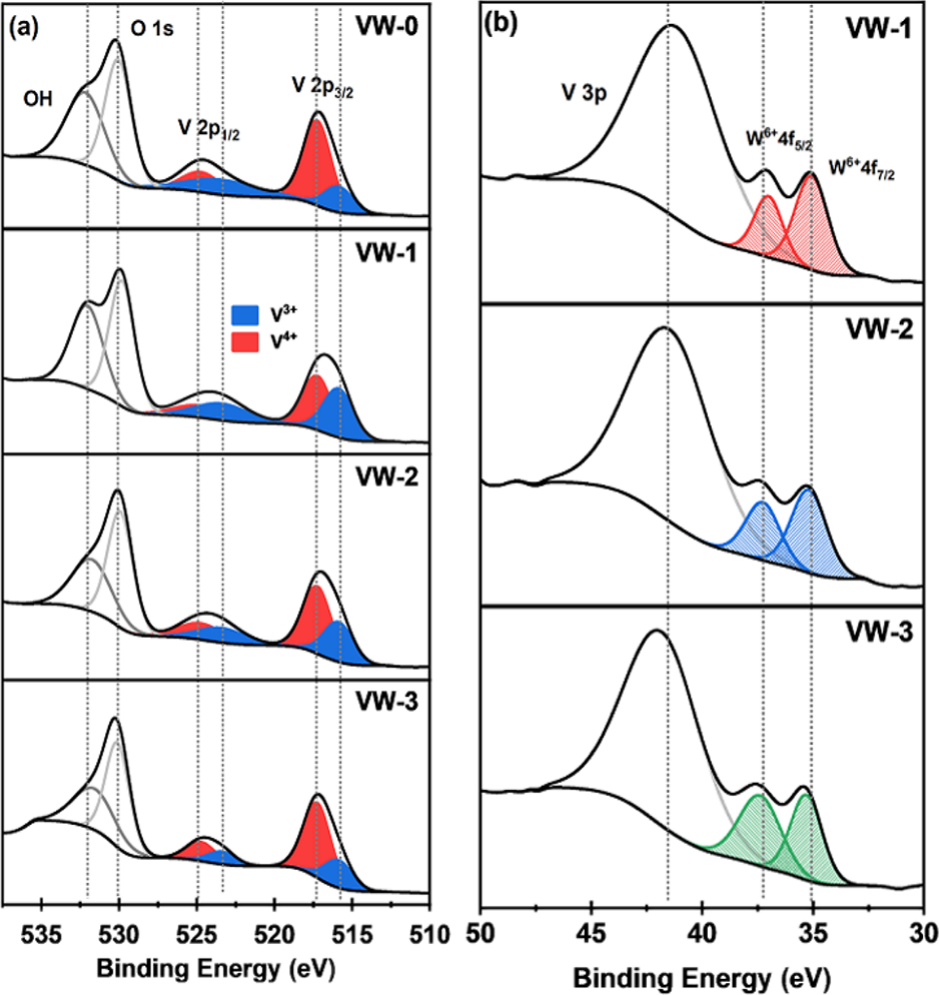
XPS spectra of pure VO_2_ and V_1–*x*_W_*x*_O_2_ thin films: (a) OH, O 1s, and V 2p and (b) V 3p and W 4f.

X-ray photoelectron spectroscopy (XPS) analysis was performed to ascertain the correct electronic states of vanadium(V) and tungsten (W) in the undoped and W-doped VO_2_ thin films. It is well-known that the valence states of V and W can significantly affect the VO_2_ thin film transition temperature.^26−28^Figure 6 shows XPS spectra of pure VO_2_ and V_1–*x*_W_*x*_O_2_ thin film samples (VW-0, VW-1, VW-2, VW-3), which were deconvoluted with peak-fitting of XPS spectral of hydroxyl (OH), oxygen (O s1), and V-2p to determine the prominent characteristics binding energies. The oxidation states of V-2p present in the thin film sample surface are composed of typical two-peak patterns of V-2p_1/2_ and V-2p_3/2_, which are attributed to the spin–orbital splitting features. The binding energies with peak positions due to spin splitting feature V-2p_3/2_, which occurred at ∼515 and ∼517 eV, are ascribed to V^3+^ and V^4+^ oxidation states of V species in pure and doped thin films,^29,^ respectively. Similarly, the spectral feature V-2p_1/2_ has corresponding binding energy peaks at ∼523 and ∼524 eV, belonging to V^3+^ and V^4+^ oxidation states.^29^ According to Kurmaev et al.,^30^ the presence of V^3+^ valence states in all the thin film samples prepared may be attributed to the high-temperature environment used during sample fabrication and oxygen vacancies, leading to thin film charge localization and surface segregation.

Meanwhile, the XPS spectral peak of O 1s appeared at ∼529 eV, which can be assigned to O^2–^ in the V–O binding, while the OH peak occurred at ∼531 eV. Liu et al.^27^ reported that the presence of oxygen vacancies in the crystal lattice had a great influence on the VO_2_ thin film transition temperature, electrical and optical properties. The spectral feature that emerged at 531.4 eV corresponds to the OH concentration, which decreases with an increase in tungsten doping concentration. The presence of the OH content on the surface of the VO2 thin film may be ascribed to the environment and surface water adsorption. XPS spectra depicted in Figure 6b show W 4f photoelectron spectra of samples VW-1, VW-2, and VW-3 with the peaks located at 35.07 and 37.13 eV confirming the existence of W 4f_7/2_ and W 4f_5/2_ induced by W^6+^ ions. The binding energy peak at 41.5 eV is ascribed to V 3p.

**Figure 7:**
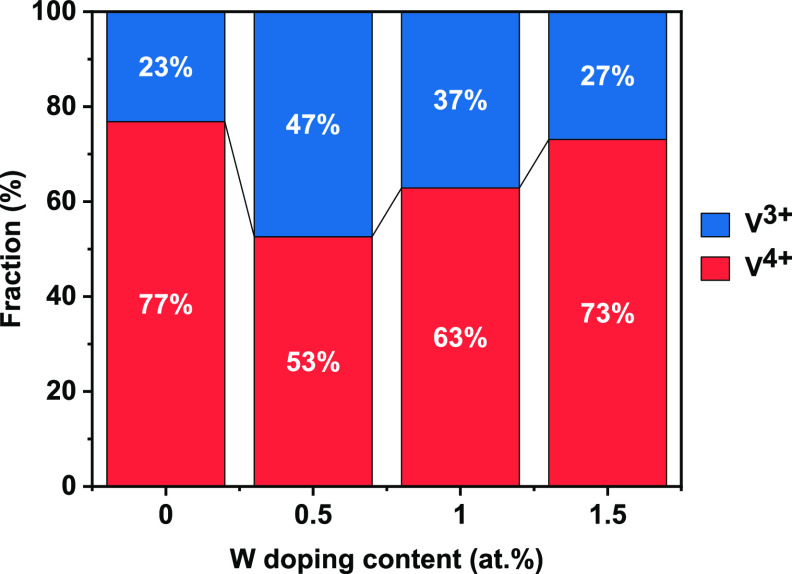
Average fraction of V^3+^ and V^4+^ contents in the thin films prepared as a function of W content.

The influence of the W content on the V valence states was investigated by fitting the area under the curves of V^3+^ and V^4+^. Figure 7 compares the V^3+^ and V^4+^ valence state content percentage ratios as a function of W doping concentration. The proportion of V^3+^ decreases, and V^4+^ increases with increasing W concentration, which confirms the stabilization of the V^4+^ state. The chemical composition of each sample prepared was determined to be VO_1.69_, VO_1.47_, VO_1.21_, and VO_1.14_ for samples VW-0, VW-1, VW-2, and VW-3. This demonstrates that oxygen deficiency increases by increasing the W content under the same fabrication condition.

### 3.5 Lattice Parameter Distortions Drove by Temperature-Dependent XRD Data

**Figure 8:**
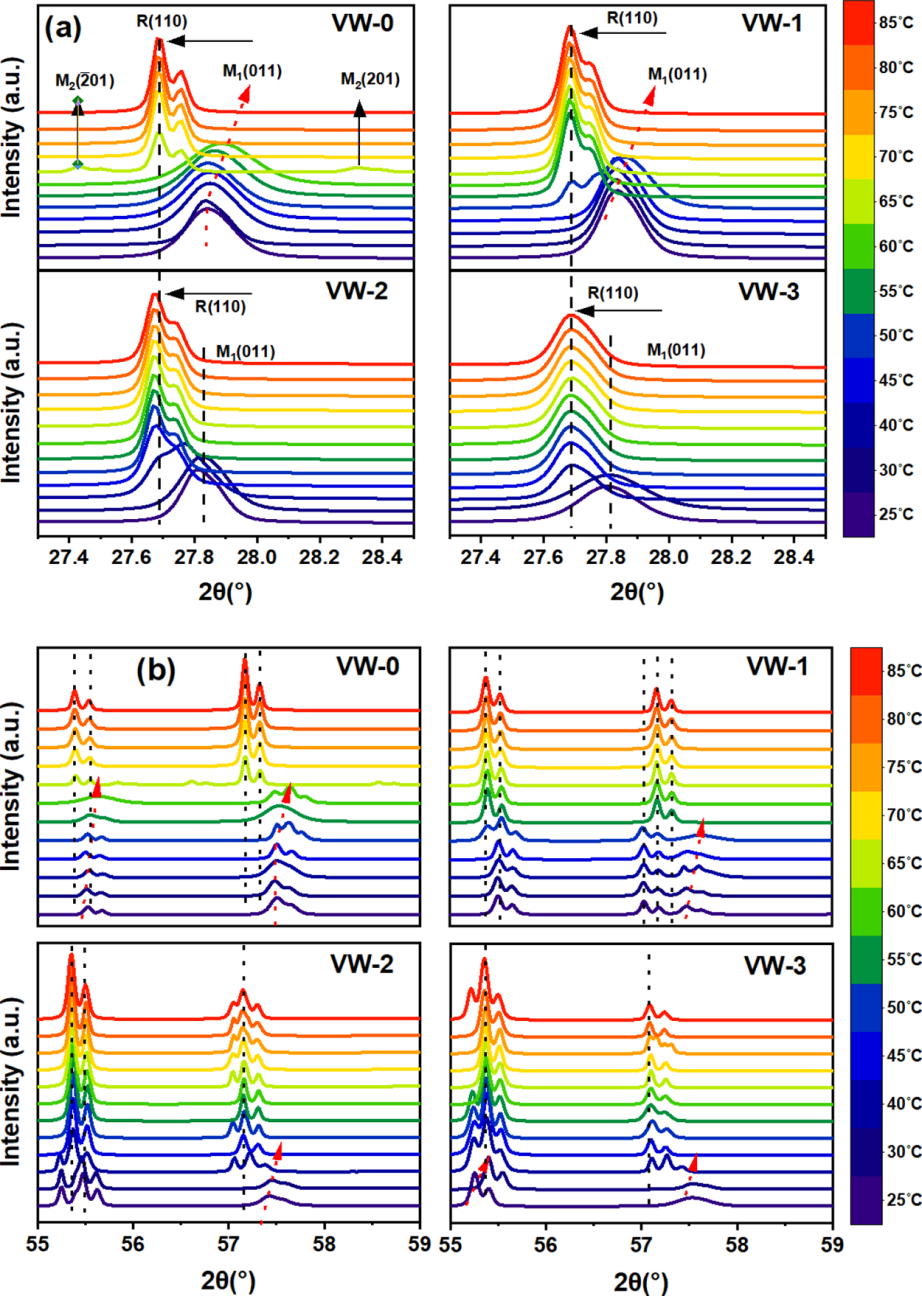
Temperature-dependent XRD patterns for samples VW-0, VW-1, VW-2, and VW-3 with heating temperatures ranging from 25 to 85 °C exhibiting phase-transition related to changes in the diffraction patterns are visible: (a) selected 2θ range of 27.4° to 28.4° and (b) selected 2θ range of 55° to 59°.

Following the observed temperature-related changes to the physical and optical properties, we investigated the VO_2_ and W^6+^ doped VO_2_ thin film microstructures by performing temperature-dependent XRD measurements. This provides a clearer quantitative understanding of how the W^6+^ content affects the VO_2_ thin film crystal structure and lattice parameters during the MIT mechanism from the M1 → M2 → R and M1 → R transition. Figure 8a illustrates 2θ scans temperature-dependent structural phase transition of the VO_2_ and W^6+^-doped VO_2_ films, with 2θ between 27.4° and 28.4° and at temperatures ranging from 25 to 85 °C covering the range over which the physical properties are changing. The diffraction peak of the VO_2_(M1) (011) phase of the thin films at a low-temperature range undergoes a change to the R(110) phase at a high temperature (JCPDS file 01–079–1655). In Figure 8a, two different transition peaks emerged from samples VW-0 and VW-1, denoted by M1(011) peaks at 27.84° and R(110) peaks at 27.68°, transitioning from room temperature (25 °C) to a high temperature of 85 °C, respectively. As the temperature increases from 25 to 60 °C and from 25 to 50 °C for samples VW-0 and VW-1, the M1 peak is shifted to the larger angle, whereas peak R(110) arises from moving between 65 and 60 °C, and continues to stabilize further above 80 °C. At the elevated temperature of around 65 °C, we observe three diffraction peaks occurring at 27.43°, 27.68°, and 28.32° labeled as M2(−201), R(110), and M2(201), which provide clear evidence for the coexistence of multiple phases in sample VW-0.^31^ Similarly, sample VW-1 reveals three diffraction peaks identical to those of sample VW-0. The M2(201) intermediate structure may be ascribed to different mechanisms, such as strain and stress at the thin film interface, doping with W^6+^ and defects on the thin film.^24^ In the case of sample VW-3, two peaks at 2θ of 27.65° and 27.84° can be attributed to the monoclinic M1 phase at a lower temperature in the presence of the metallic R phase at a higher temperature.

Furthermore, Figure 8b shows XRD patterns between 2θ of ∼55° and ∼59° obtained while heating the VO_2_ and W^6+^-doped VO_2_ thin film samples. The VO_2_ diffraction peaks occur at ∼55.43° (220) and ∼57.8° (022) and are also shifted to the higher angle at the low-temperature range, corresponding to the monoclinic M1 phase. At the elevated temperature, the XRD patterns move to lower angles, indicating a phase transition from monoclinic M1 to the metallic R phase as a result of the heating process. Meanwhile, shifting the M1 structural phase to a higher 2θ angle during heating results from the strain induced at the thin film and silica substrate interface, leading to a mesoscopic phase separation. These results demonstrate that the various diffraction peaks seen in the VO_2_ and W^6+^-doped VO_2_ thin films fabricated by fs-PLD can be used to predict VO_2_ (M1) phase transition temperature accurately.

**Figure 9:**
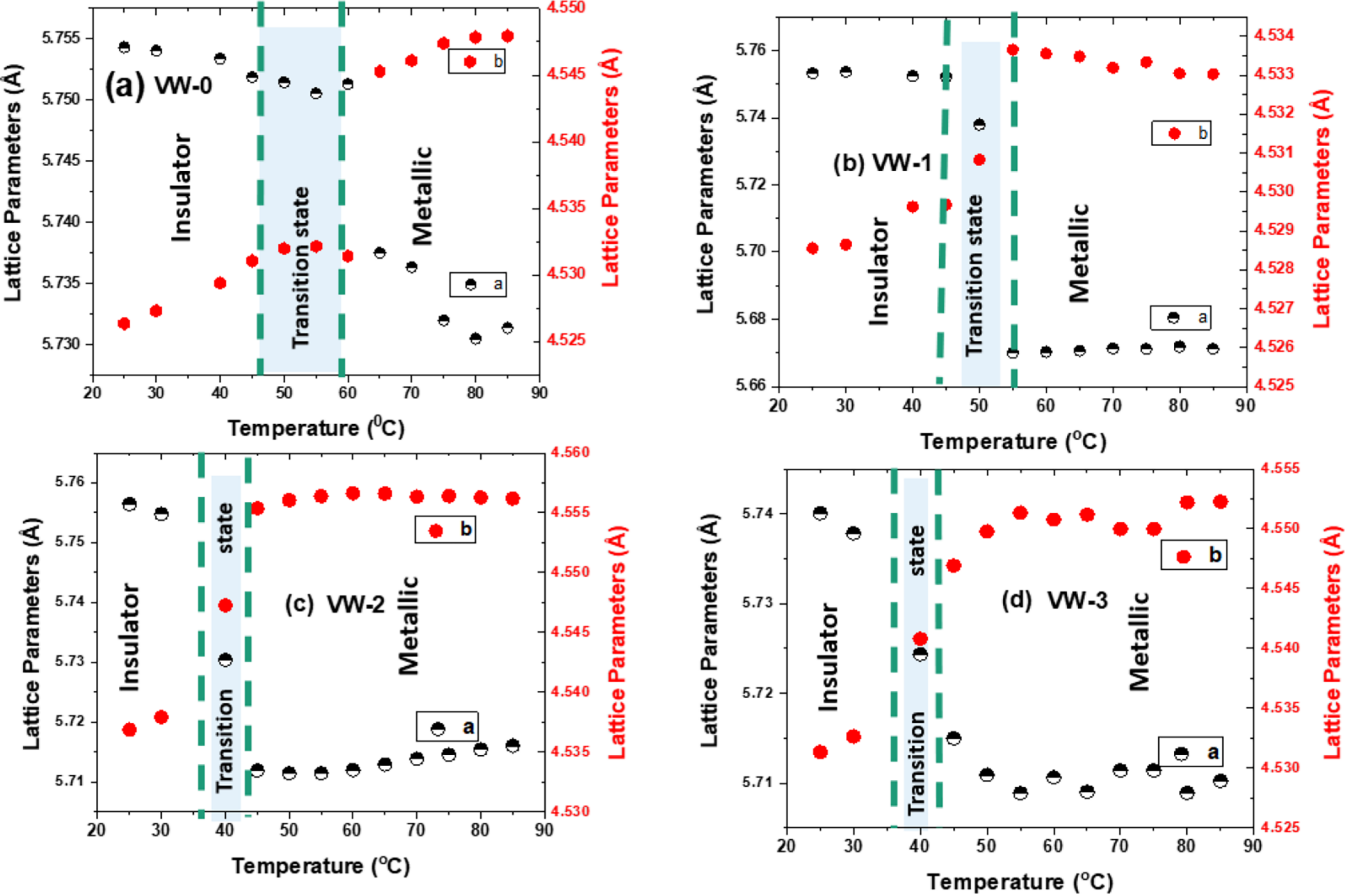
Lattice parameters *a* and *b* of VO_2_ and W-VO_2_ thin films as function temperature (a) VW-0, (b) VW-1, (c) VW-2 and (d) VW-3.

The local crystalline lattice parameters *a* and *b* were calculated using FullProf software to help shed light on the subsequent measured behavior. The trend of *a* and *b* lattice parameters as a function of temperature was obtained by using temperature dependent XRD patterns in the range of 2θ from 26° to 60°. Figure 9 illustrates a plot of the variation of these lattice parameters *a* and *b* with temperature for samples VW-0, VW-1, VW-2, and VW-3 exhibiting hysteresis properties, respectively. A-lattice (*b*-lattice) gradually shifted to a lower (higher) value as the temperature increased, with a clear distinction between the insulator state (M1) and metallic state (R). This trend indicates that *a*-lattice and *b*-lattice parameters are associated with contraction and expansion in the VO_2_ thin film samples during heating from room temperature up to 85°. The range of the *a* and *b* lattice parameters seen in samples VW-0, VW-1, VW-2, and VW-3 are in good agreement with the observations by Liu et al.,^15^ who deposited VO_2_ thin films on (0001)-Al_2_O_3_ single-crystal substrates using RF magnetron sputtering. Similarly, the trends of the *a* and *b* lattice parameters as a function of temperatures are comparable to our results.

**Figure 10:**
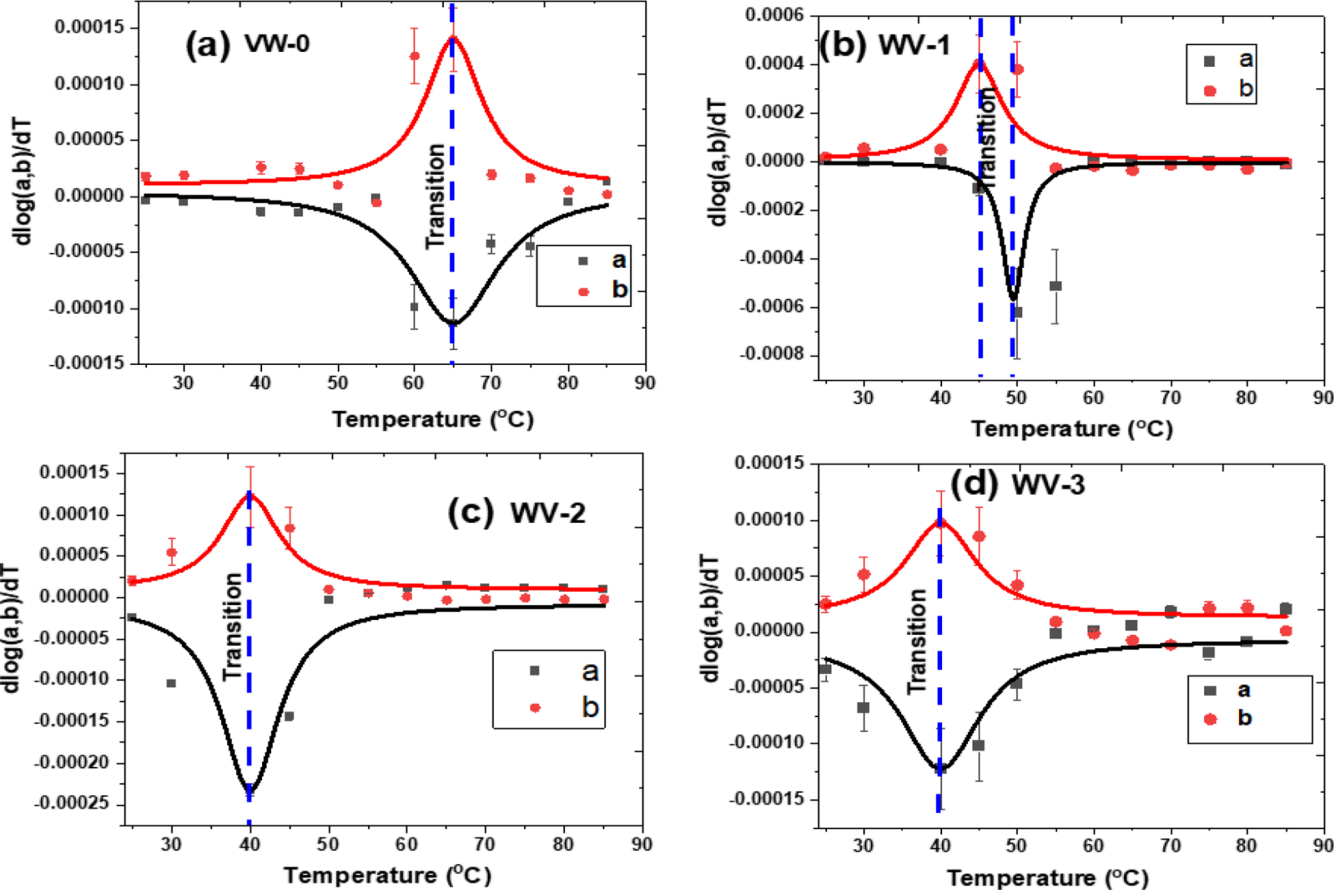
First derivative of the log_10_(*a* & *b* lattice parameters) as a function of temperature fitted with Lorentz equation: (a) WV-0, (b) WV-1, (c) WV-2, and (d) WV-3.

The MIT transition temperatures of the pure VO_2_ and W^6+^-doped VO_2_ thin films were evaluated by employing the first derivative logarithms of lattice parameters *a* and *b* for the temperature {i.e. d[log(*a*&*b*)]/d*T*}. Figure 10a–d shows the plots of d[log(*a*&*b*)]/d*T* versus temperature, which was fitted with the Lorentz equation using OriginPro software. The phase transition temperatures (*T*_t_) of the thin films were determined using the expression . Table 1 below summarizes the phase transition temperatures of various samples during the contraction and expansion of the *a* and *b* parameters. It is observed that the average transition temperature of samples VW series decreases from ∼66 to 38 °C as the W^6+^ concentration increases from 0.0 to 1.5 wt %. Thus, such a decrease in phase transition is mostly attributed to an increase in W^6+^ doping concentration, induced microstrain and particle sizes, as illustrated in Figure 5c. In addition, the VO_2_ thin film induces compressive strain along the *a* axis, which can lower the transition temperature to near room temperature, as shown in Figure 10a–d. The structural phase transition temperatures obtained from samples VW-0, VW-1, VW-2, and VW-3 correlate with the results by Chen et al.,^5^ where they synthesized W^6+^-doped VO_2_ thin film samples with W^6+^ concentrations of 0%, 0.5%, 1%, 1.5%, and 2% using a cosputtering method and followed by post-annealing. They measured temperature-dependent transmission in the near-infrared region and reported tuning the phase transition temperatures from 64.3 to 36.5 °C. Similarly, Rajeswaran et al.^24^ fabricated polycrystalline W_*x*_V_1–*x*_O_2_ thin films using ultrasonic nebulized spray pyrolysis of aqueous combustion mixtures, with W^6+^ content varying between *x* = 0.2 and 2.0 at. %. The authors reported that transition temperatures decreased from 68 to 25 °C by doping the VO_2_ with 2.0 at% of W^6+^ and measuring the temperature-dependent resistance of the thin films. According to these literature results, the variation in the transition temperature is affected by the nature of the VO_2_ thin film phases, such as M1, M2, T, and R, together with surface morphology and orientation of the grains and their grain boundaries.^32^ According to Tang et al.^23^ and He et al.^33^ the loss of direct bonding between the V^4+^–V^4+^ homopolar and V^3+^–V^4+^ heteropolar bonds by doping W^6+^ with VO_2_ destabilizes the VO_2_ semiconducting phase to lower the phase transition temperature. In addition, a high doping concentration of the W^6+^ valence state may lead to a boost of free-electron concentration and then lead to a transition temperature drop.^34^ It is also important to note that the transition temperatures obtained from our study are comparable to temperature-dependent resistivity transition temperatures of similar doping concentrations reported elsewhere.^35,36^

**Table 1:** *a* and *b* Lattice Parameters Transition Temperatures and Average Transition Temperature of the As-Deposited VO_2_ and W^6+^-Doped VO_2_ Thin Films

sample ID	transition temp. for a-lattice (°C) (*T*_a_)	transition temp. for b-lattice (°C) (*T*_b_)	average transition temperature (°C) (*T*_t_)
VW-0	65.3 ± 4.4	65.7 ± 5.8	65.5 ± 5.1
VW-1	45.7 ± 3.6	49.5 ± 4.3	47.6 ± 3.9
VW-2	40.9 ± 2.7	39.9 ± 1.6	40.4 ± 2.2
VW-3	38.4 ± 1.8	38.4 ± 3.8	38.4 ± 2.8

### 3.6 Temperature-Dependent Electrical Resistivity of VO_2_ Phase Transition

**Figure 11:**
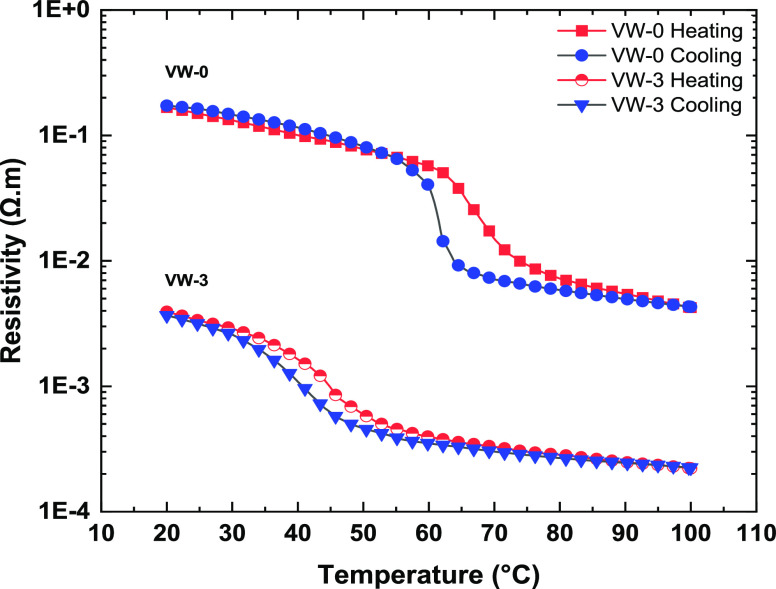
Resistivity as a function of temperature curve of samples VW-0 and VW-3.

**Figure 12:**
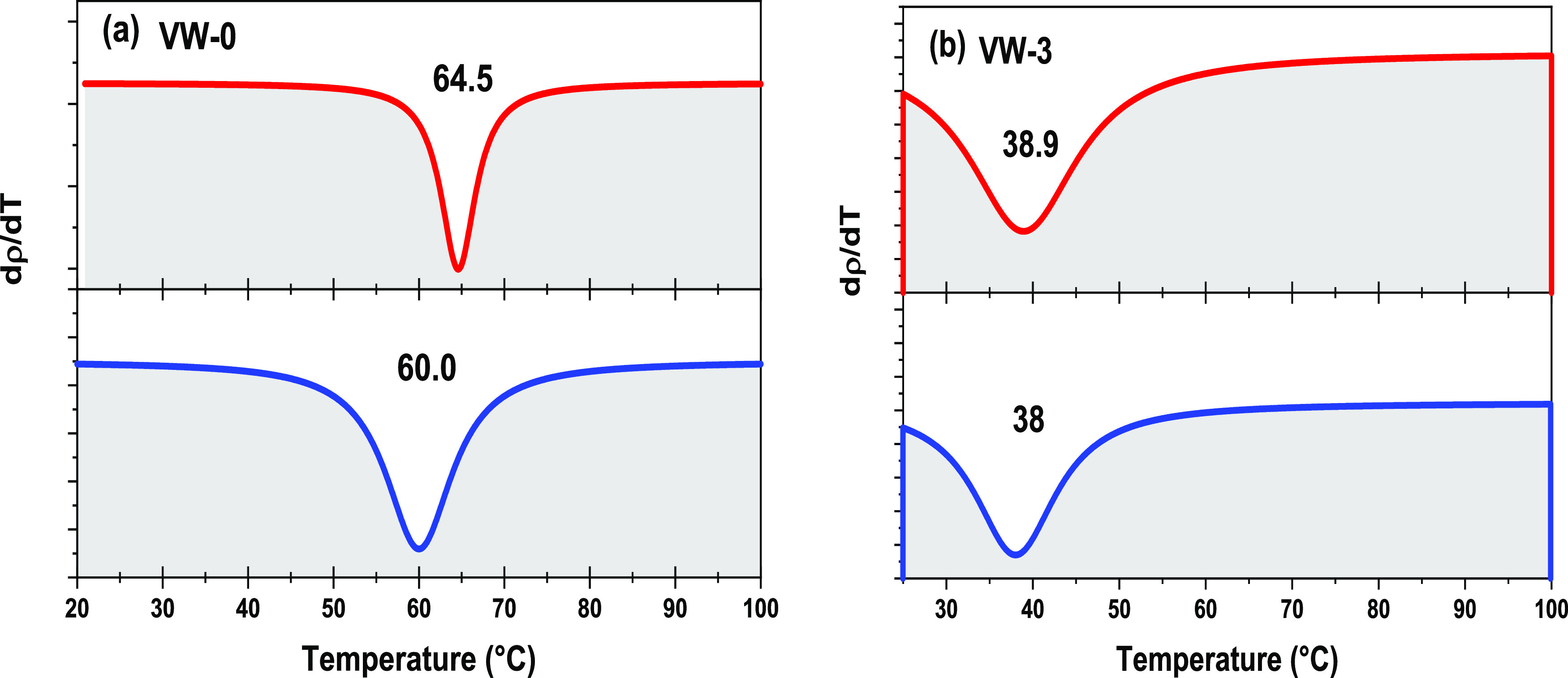
Gaussian fitting of the first derivative of the resistivity with respect to temperature vs temperature for samples (a) VW-0 and (b) VW-3.

The temperature-dependent resistivities of the thin films prepared were investigated by using a four-point probe purchased from Ossila Ltd. The electrical resistivity was recorded from room temperature to 100 °C for comparison with the temperature-dependent XRD results illustrated in Figures 9 and 10. Figure 11 shows the results of temperature-dependent electrical resistivity plots during the heating and cooling cycles of samples VW-0 and VW-3. The thin film sample VW-0 exhibits a metal-to-insulator transition with 2 orders of magnitude change in resistivity switched compared to sample VW-3, which has a resistivity change by a single order. The semiconductor metal-to-insulator transition was determined utilizing the first derivative of the resistivity with respect to temperature [ i.e., d[log(ρ)]/d*T*]. The resulting curves are shown in Figure 12a,b for samples VW-0 and VW-3, which are fitted with Gaussian functions with minima corresponding to heating, *T*_h_, and cooling, *T*_c_ phase transition temperature. Similar temperature-dependent resistivity measurements were performed for samples VW-1 and VW-2 to determine the transition temperature, which is not shown (to be published later). Table 2 represents the average transition temperatures obtained from temperature-dependent resistivity measurements, which are in agreement with those reported from the *a* and *b* lattice parameters presented in section 3.5. The average MIT decreases with an increasing doping concentration of W.

**Table 2:** Average Transition Temperature Obtained from Heating and Cooling Temperature-Dependent Resistivity Measurements for Undoped VO_2_ and All W-Doped VO_2_ Thin Films

sample ID	transition temp. for heating (°C) [*T*_*h*_]	transition temp. for cooling (°C) [*T*_*c*_]	average transition temperature (°C)
VW-0	64.5 ± 2.7	60.0 ± 2.5	62.3 ± 2.6
VW-1	46.8 ± 3.2	47.8 ± 3.3	47.3 ± 3.2
VW-2	41.0 ± 1.5	42.0 ± 2.3	41.5 ± 1.9
VW-3	38.9 ± 1.3	38.0 ± 2.7	38.5 ± 2.0

## 4 Conclusions

A high repetition rate femtosecond-PLD approach has been used to deposit thicker VO_2_ and W^6+^ doped VO_2_ on silica substrates. The thin films’ surface morphology, particle size, and crystal orientation were confirmed using SEM and room temperature XRD measurements. The XRD measurements revealed mixed phases of the highly dense polycrystalline monoclinic crystalline structures of VO_2_(M1) and (M2) for W^6+^-doped VO_2_ thin film samples. With increasing W^6+^ concentration, the VO_2_(M2) phase becomes dominant and stable and exists together with VO_2_(M1) and VO_2_(B) phases; however, it suppresses the XRD peak intensity of the VO_2_(M1) phase due to the W^6+^ content. Thus, this is ascribed to the strain induced by doping the VO_2_ with the W^6+^ ions and the uniformly distributed W^6+^ in the VO_2_ matrix, favoring the VO_2_(M2) phase formation instead of the VO_2_(M1) phase. The temperature-dependent measurements showed a remarkably sharp change in the *a* and *b* lattice parameters from room temperature to a high temperature of about 85 °C. These lattice parameter changes result in a sharp decrease at the MIT temperature, corresponding to the structural phase transformation from monoclinic M1 to the metallic R phase. The phase transition temperature decreases from ∼66 to 38 °C when increasing the W^6+^ concentration. This study demonstrates the nature of the changes in the temperature-dependent lattice parameters, offering the potential to understand and more accurately predict the structural phase transitions of VO_2_ and W^6+^-doped VO_2_ thin films, which affect the resistivity and optical transmission behavior as a function of temperature.
